# SP600125 Attenuates Nicotine-Related Aortic Aneurysm Formation by Inhibiting Matrix Metalloproteinase Production and CC Chemokine-Mediated Macrophage Migration

**DOI:** 10.1155/2016/9142425

**Published:** 2016-09-01

**Authors:** Zhen-Zhen Guo, Qun-An Cao, Zong-Zhuang Li, Li-Ping Liu, Zhi Zhang, Ya-Juan Zhu, Guang Chu, Qiu-Yan Dai

**Affiliations:** ^1^Department of Cardiology, Shanghai General Hospital, Shanghai Jiao Tong University School of Medicine, Shanghai 200080, China; ^2^Department of Cardiology, Guizhou Provincial People's Hospital, Guiyang 550002, China; ^3^Department of Cardiology, Yancheng First People's Hospital, The Fourth Affiliated Hospital of Nantong Medical University, Jiangsu 224001, China

## Abstract

Nicotine, a major chemical component of cigarettes, plays a pivotal role in the development of abdominal aortic aneurysm (AAA). c-Jun N-terminal kinase (JNK) has been demonstrated to participate in elastase-induced AAA. This study aimed to elucidate whether the JNK inhibitor SP600125 can attenuate nicotine plus angiotensin II- (AngII-) induced AAA formation and to assess the underlying molecular mechanisms. SP600125 significantly attenuated nicotine plus AngII-induced AAA formation. The expression of matrix metalloproteinase- (MMP-) 2, MMP-9, monocyte chemoattractant protein- (MCP-) 1, and regulated-on-activation, normal T-cells expressed and secreted (RANTES) was significantly upregulated in aortic aneurysm lesions but inhibited by SP600125.* In vitro*, nicotine induced the expression of MCP-1 and RANTES in both RAW264.7 (mouse macrophage) and MOVAS (mouse vascular smooth muscle) cells in a dose-dependent manner; expression was upregulated by 0.5 ng/mL nicotine but strongly downregulated by 500 ng/mL nicotine. SP600125 attenuated the upregulation of MCP-1 and RANTES expression and subsequent macrophage migration. In conclusion, SP600125 attenuates nicotine plus AngII-induced AAA formation likely by inhibiting MMP-2, MMP-9, MCP-1, and RANTES. The expression of chemokines in MOVAS cells induced by nicotine has an effect on RAW264.7 migration, which is likely to contribute to the development of nicotine-related AAA.

## 1. Introduction 

Abdominal aortic aneurysm (AAA) is an important cause of mortality in older adults. It affects approximately 5% of men and 1% of women over the age of 60, and it is the thirteenth leading cause of death in the USA [1]. AAA is a focal full-thickness dilatation of the abdominal aorta to greater than 1.5 times its normal diameter. The diameter of the aortic aneurysm is often used to assess the risk of rupture. Currently, therapeutic strategies for the treatment of AAA development and rupture are limited to surgical treatments, which are clinically suitable for large but not for small aneurysms; therefore, an effective nonsurgical therapy to attenuate the formation and development of AAA is necessary.

Smoking is the only modifiable risk factor associated with the formation, development, and rupture of AAA. Nicotine, as a major chemical component of cigarette smoke, receives increasing research attention [2, 3]. Recent studies indicated that infusion of nicotine (1.5 mg/kg/d or 5 mg/60 d) markedly increased the incidence of AAA in apolipoprotein E knockout (ApoE^−/−^) mice [4, 5]. Another study has shown that AngII plays an important role in the development of aortic aneurysms, with a low dose of AngII (0.72 mg/kg/d) inducing a low incidence of AAA [6]. But the existing animal model is not ideal enough to simulate the pathological changes in the older smoker; the deficiency of apolipoprotein E may influence the formation of AAA. To identify the pathological features triggered by nicotine, in this study, we coadministered nicotine (4 mg/kg/d, a dose producing a plasma concentration in rodents similar to that in heavy smoker [7]) and a low dose of AngII into aged C57BL/6J wild-type mice by infusion.

AAA is characterized by chronic inflammation, mainly involving mononuclear phagocytes and lymphocytes [8]. Inflammatory cells infiltrating the aortic wall are thought to be important in the destructive connective tissue remodeling that occurs before and during aneurysmal degeneration, particularly through the production of proinflammatory cytokines and enzymes capable of degrading elastin, collagen, and other structurally important matrix proteins. Despite the recognized association between chronic inflammation and aneurysm degeneration, it is notable that several chemotactic cytokines with activities towards monocytes, macrophages, and lymphocytes are produced in human AAA tissue, including the CC chemokines MCP-1 and RANTES. Expression of these chemokines has been confirmed as an early event during the progression of experimental aneurysm formation by promoting leukocyte infiltration into the outer aortic wall in an elastase-induced AAA animal model [9].

c-Jun N-terminal kinase (JNK), a member of the mitogen-activated protein kinases (MAPK) family, plays a role in multiple processes such as inflammation, proliferation, and apoptosis. The JNK signaling pathway has been shown to contribute to the development of aortic aneurysm. Inhibition of JNK attenuated aortic aneurysm formation in an elastase-induced AAA model and led to a reduction in the aortic diameter after the establishment of AAA [10]. Meanwhile, the activation of JNK can upregulate MMPs in smooth muscle cells and, subsequently, AAA formation [11]. While these studies have demonstrated the importance of JNK in the formation of aortic aneurysm, the relationship between JNK and nicotine-related abdominal aortic aneurysm requires further research.

**Figure 1:**
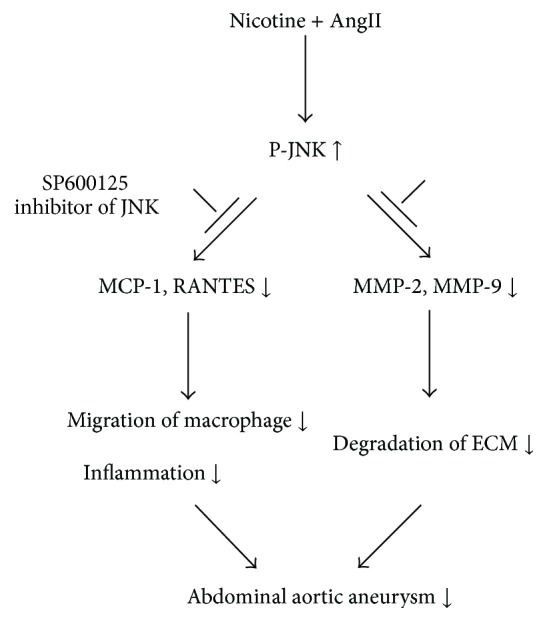
Proposed mechanism of SP600125-mediated suppression of nicotine-related AAA in the C57BL/6J mouse model. AngII, angiotensin II; JNK, c-Jun N-terminal kinase; MCP-1, monocyte chemoattractant protein-1; RANTES, regulated-on-activation, normal T-cell expressed and secreted; MMP, matrix metalloproteinase; ECM, extracellular matrix.

In the present investigation, we focused on MCP-1 and RANTES, whose roles in vascular biology have been characterized. The purpose of this study was to investigate the potential roles of CC chemokines in aneurysmal degeneration. Accordingly, we examined whether (1) the development of nicotine plus AngII-induced AAA in the mouse is accompanied by altered local production of MCP-1 and RANTES under control of the JNK pathway* in vivo* and (2) exposure of cultured MOVAS cells to nicotine has an effect on their cellular chemokine expression, which may induce the migration of RAW264.7 cells. The results of our experiments would provide evidence that nicotine-induced production of CC chemokines within the aortic media may be an important biologic mechanism underlying experimental aneurysm degeneration (Figure 1). Meanwhile, AAA has a tight relationship with vascular remolding, in which MMPs play a pivotal role, and MMP-2 and MMP-9 are considered to be crucial to extracellular matrix (ECM) degradation (Figure 1); furthermore, MMPs may facilitate inflammation [12] and vice versa, inflammation contributions to extracellular matrix remodeling [13]. Accordingly, we evaluated the production of these two MMPs in the urine aneurysm model treated with nicotine plus AngII.

## 2. Materials and Methods

### 2.1. Animal Experiments

This research was approved by the Animal Care and Use Committee, School of Medicine, Shanghai Jiao Tong University, and the procedures in the Principles of Laboratory Animal Care and Guidelines for the Care and Use of Laboratory Animals were followed. One-month-old male C57BL/6J wild-type mice were purchased from the Model Animal Research Center of Nanjing University. The mice were maintained in cabin-type isolators under standard environmental conditions: 22–25°C and 40–70% humidity, with a 12-h photoperiod. The mice were given free access to a sterilized NIH-31 modified mouse diet and water. Ten-to-twelve-month-old mice were used in the experiments. The chemicals used in the animal experiments were obtained from Sigma-Aldrich (St. Louis, USA).

The animal experiments comprised two parts. In the first part, 42 mice were randomly divided into 4 treatment groups: (1) a saline-infused group (control group; *n* = 10); (2) a nicotine-treated group (*n* = 10); (3) an AngII-treated group (*n* = 10); and (4) a cotreatment group (nicotine plus AngII; *n* = 12), as described previously [14]. Both nicotine and AngII were dissolved in 0.9% saline and delivered at 4 mg/kg/d and 0.72 mg/kg/d, respectively. Miniature osmotic pumps (Alzet, Model 2004; DURECT, Cupertino, CA, USA) containing 0.9% saline, AngII, nicotine, or nicotine plus AngII were implanted subcutaneously for 28 days.

On the basis of former research, in the second part, another 40 mice were randomly divided into 2 groups: (1) a cotreatment group (nicotine plus AngII; *n* = 20) and (2) a cotreatment plus SP600125 group (*n* = 20). Nicotine and AngII were delivered as mentioned above. SP600125 was dissolved in DMSO and delivered via hypodermic injection at 30 mg/kg, twice per day. As a control, mice were injected with DMSO.

At the end of the 28-day treatment period, the mice were euthanized by cervical vertebra dislocation. The aortas of euthanized mice were observed and dissected under a surgical microscope (SXP-1C; Shanghai Medical Instruments, Shanghai, China) at the end of the 28-day treatments. Digital photographs of the abdomens were taken to measure the maximum external diameters. A segment of the suprarenal abdominal aorta (±5 mm in length) as the site with the highest risk of AAA was excised from each mouse and fixed in 10% neutral formalin for histological analysis. The remaining aortic segment was snap-frozen in liquid nitrogen and stored at −80°C for western blotting and quantitative reverse transcription polymerase chain reaction (qRT-PCR) analysis.

### 2.2. Immunohistochemistry

Cross sections (4 *μ*m thick) of the suprarenal abdominal aorta tissue were used for histological analysis. The aortic cross sections were deparaffinized and hydrated in order, then rinsed 3 times with tap water, and hyperbaric heating antigenic repair for 3–5 min, and then rinsed with several changes of PBS, followed by blocking with 5% goat serum in PBS for 30 min. Slides were incubated with rabbit anti-mouse RANTES immunoglobulin G (IgG), rabbit anti-mouse MCP-1 IgG, rabbit anti-mouse MMP-2, rabbit anti-mouse MMP-9, or reagent-grade nonimmune rat IgG (R&D Systems, Minneapolis, MN, USA) overnight at 4°C in a humidified chamber. Then, the slides were incubated with biotin-conjugated goat anti-rabbit IgG (1 : 200 in PBS; Vector Laboratories, Burlingame, CA, USA) for 30 min at room temperature, followed by incubation with the horseradish peroxidase-conjugated avidin/biotin complex for 10 min. Immune complexes were detected with horseradish peroxidase substrate (Vector Laboratories, Burlingame, CA, USA). Sections were counterstained with hematoxylin before examination with light microscopy.

### 2.3. Culture of Mouse Aortic Vascular Smooth Cells and Macrophages

Mouse vascular smooth muscle (MOVAS, ATCC, Manassas, VA, USA) cells and mouse macrophage cells (RAW264.7, ATCC, Manassas, VA, USA) were cultured in high-glucose Dulbecco's Modified Eagle Medium (DMEM, Hyclone, Logan, UT, USA) supplemented with 10% fetal bovine serum (FBS, Gibco Invitrogen, Grand Island, NY, USA) in a humidified atmosphere of 95% air and 5% CO_2_ at 37°C. The medium was changed every 2 days. The cells were plated on 6-cm diameter culture dishes at a density of 2 × 10^5^ cells/mL. Approximately 48 h later, when most of the cells were in the logarithmic growth period, the medium was changed to serum-free medium to starve the cells overnight. Then, the medium was changed to fresh DMEM without FBS, and the cells were pretreated with the JNK inhibitor SP600125 (Sigma-Aldrich, St. Louis, MO, USA) for 1 h prior to being supplemented with indicated concentrations of nicotine for 3 h. Both the supernatant and the cells were harvested for subsequent experiments.

### 2.4. RNA Extraction and qRT-PCR

Total cellular RNA was extracted using TRIZOL reagent (Invitrogen, Carlsbad, CA, USA) according to the manufacturer's instruction and reverse-transcribed using a reverse transcription kit (Takara, Otsu, Japan). The MCP-1 and RANTES mRNA expression levels were detected by qRT-PCR using SYBR green dye. qRT-PCR was carried out using the ABIPRISM 7000 sequence detection system (Applied Biosystems, Foster City, CA, USA). Expression of the genes of interest was normalized to GAPDH expression. All values were expressed as fold changes relative to GAPDH expression. The cycle threshold (Ct) for each sample was calculated, with the relative expression calculated as the difference (ΔΔCt) between the ΔCt values of the test sample and those of the control sample. The relative expression of target genes was calculated and expressed as 2^−ΔΔCt^. Dissociation curves were utilized to confirm the absence of significant amounts of primer dimers.

### 2.5. Western Blotting

Total protein of aortic issue was extracted with RIPA lysis buffer (Beyotime Institute of Biotechnology, Haimen, China) with 100 *μ*g/mL phenylmethylsulfonyl fluoride (Roche, Molecular Biochemicals, Mannheim, Germany) added according to the manufacturer's protocol. A BCA protein assay kit (Beyotime Institute of Biotechnology, Haimen, China) was used to quantify the total protein. Aliquots of protein were separated by 8% sodium dodecyl sulfate-polyacrylamide gel electrophoresis and transferred to Immobilon-P polyvinylidene membranes (Bio-Rad Laboratories, Hercules, CA, USA). Membranes were blocked in 5% nonfat milk/TBST (25 mM Tris-HCl, 150 mM NaCl, 0.1% Tween-20; pH 7.4) for 1 h and incubated with rabbit polyclonal antibodies against MCP-1 (1 : 1000, Abcam, Cambridge, UK), RANTES (1 : 500, Abcam, Cambridge, UK), MMP-2 (1 : 500, Abcam, Cambridge, UK), or MMP-9 (1 : 500, Abcam, Cambridge, UK) in primary antibody dilution solution (Beyotime Institute of Biotechnology, Haimen, China) overnight with gentle shaking. The next day, the membranes were washed with TBST for 30 min at room temperature, followed by incubation with HRP-conjugated secondary antibodies for 1 h and three washes in TBST. Then, the membranes were incubated with rabbit monoclonal antibody against GAPDH (1 : 1000, Cell Signaling Technology, Boston, MA, USA) overnight with gentle shaking, probed with HRP-conjugated goat anti-rabbit secondary antibody (1 : 2000, Cell Signaling Technology, Boston, MA, USA) for 1 h, and washed in TBST. All membranes were visualized using the West-Pico ECL kit (Pierce, Rockford, IL, USA). Protein expression was normalized to GADPH levels.

### 2.6. Enzyme-Linked Immunosorbent Assay (ELISA)

Cell culture supernatants and serum were collected after centrifugation at 800 ×g at 4°C for 5 min. The supernatants and serum samples were aliquoted, snap-frozen, and stored at −80°C for further analysis. Humoral-response levels of MCP-1 and RANTES levels were assessed using the Mouse MCP-1 and RANTES ELISA kits (R&D Systems, Minneapolis, MN, USA) according to the manufacturer's protocols.

### 2.7. Transwell Migration Assay

Cell migration was quantitated in duplicate by use of 24-well Transwell inserts with polycarbonate filters (8-mm pore size) (Corning Costar, Acon, MA, USA). For the migration assay, 8 × 10^4^ MOVAS cells were transferred onto the membranes of the lower chambers containing DMEM with 10% fetal bovine serum. The cells were permitted to adhere in complete medium for 24 h after which the medium was changed to serum-free medium. Macrophages (1.5 × 10^5^) were added to the upper chambers of the inserts containing 200 *μ*L of high-glucose DMEM with 10% FBS. RAW264.7 cells were seeded onto the membranes of the upper chambers containing serum-free DMEM. After incubation for 8 h, the cells in the bottom chambers were quantified by light microscopy; the membrane was washed twice in phosphate-buffered saline (PBS) and fixed with methanol for 10 min. Finally, the chambers were stained with 0.5% crystal violet solution for 30 min, and the cell numbers were determined at magnifications of 10x and 400x for each well using ImageJ. The average number of cells per field of view at a magnification of 400x served as a migration index.

### 2.8. Statistical Analysis

Data were expressed as the means ± standard deviations (SDs). Data were analyzed by analysis of variance (ANOVA) followed by Bonferroni posttests or by using the Student *t*-test (for ELISA data). *P* < 0.05 was regarded as denoting statistical significance. Statistical analysis was conducted using the GraphPad InStat software (GraphPad Software, Inc., San Diego, CA, USA).

## 3. Results

### 3.1. Nicotine Plus AngII Induces Aneurysm Development

**Figure 2:**
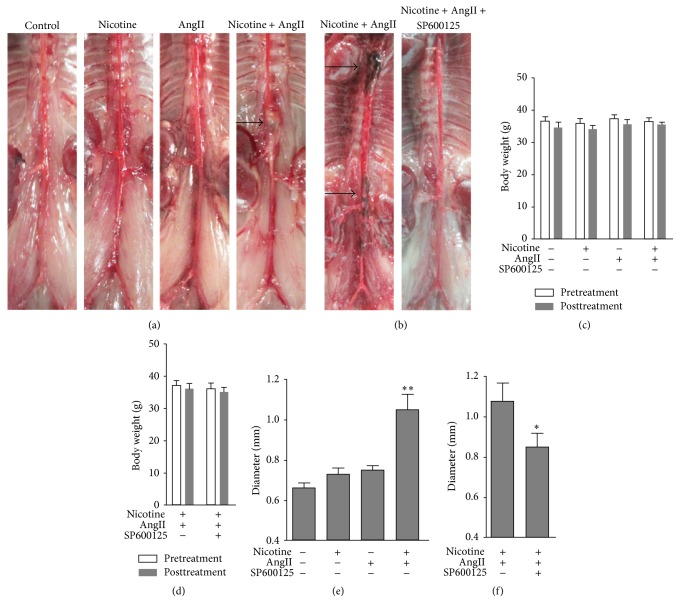
SP600125 attenuates the formation of aortic aneurysm induced by nicotine plus AngII. (a) Representative photomicrographs of abdomens of mice of all four experimental groups. No aortic aneurysms were detected in saline-, nicotine-, or AngII-treated groups. Nicotine plus AngII induced the formation of aortic aneurysms (published in Experimental & Clinical Cardiology [14]). (b) Representative photomicrographs of abdomens of the mice in the cotreatment and SP600125-supplemented groups. Nicotine plus AngII induced the formation of aortic aneurysms. No aortic aneurysms were detected in the SP600125 group. (c, d) In all experimental groups, the body weight did not change from initiation to the end of the experiment. (e, f) The maximum diameter of the suprarenal abdominal aorta was measured using ImageJ and normalized to the body surface area, which was calculated using the Meeh–Rubner formula (area = *K*(*W* ^∧^(2/3))/1000, *W*: weight, *K* = 9.1). Data are represented as the mean ± SEM. ^*∗*^ *P* < 0.01 versus nicotine or AngII group; *∗∗* refers to *P* < 0.01 versus control group. AAA, abdominal aortic aneurysm.

In the first part of our study, 42 10–12-month-old mice were infused with saline, nicotine, AngII, or nicotine plus AngII for 28 days. No changes in body weight were observed in the experimental and control groups over the course of the experiment (*P* > 0.05, Figure 2(c)) [14]. Nicotine and AngII infusions alone failed to induce macroscopic aortic aneurysm formation (Figure 2(a)). Nicotine plus AngII cotreatment induced the formation of aortic aneurysms in 6 out of 12 mice (Figure 2(a)), two of which died on the 7th and 11th day after implantation of the pump. Autopsy indicated that the two mice had died of aneurysm rupture. While no significant difference in the external diameter of the suprarenal abdominal aorta was detected between the nicotine and AngII treatment groups (*P* > 0.05), when compared to the control group, both treatments contributed to an increased maximum external diameter (Figure 2(e)). However, the maximum external diameter of the suprarenal abdominal aorta in the cotreatment group was significantly larger than those in the three other experimental groups (*P* < 0.01 versus nicotine or AngII group; *P* < 0.001 versus control group) (Figure 2(e)) [14]. Taken together, these results indicated that nicotine plus AngII contributes to the formation and development of aortic aneurysms in aged male C57BL/6J wild-type mice and even can induce early death caused by aneurysm rupture.

### 3.2. SP6000125 Attenuates AAA Formation Induced by Nicotine Plus AngII

Our findings indicated that combined loading of nicotine and AngII accelerated AAA formation in aged mice and phosphorylated JNK is significantly upregulated in aneurysm tissue, although the underlying mechanism remains elusive [14]. Therefore, we investigated whether the JNK pathway plays a role in nicotine plus AngII-induced AAA. In the second phase of the present study, nicotine plus AngII produced aortic aneurysm in 8 out of 20 mice (Figure 2(b)), approximating the incidence in our previous study [14]. Cotreatment with SP600125 significantly reduced the AAA formation induced by nicotine plus AngII (Figure 2(b)); aortic aneurysm formation was not observed in any of the 20 mice. As expected, the diameter of the abdominal aorta was markedly enlarged by cotreatment. However, the aortic diameter in SP600125-supplemented mice was significantly lower than that in the cotreated group (Figure 2(f)), suggesting that the inhibition of JNK suppresses AAA formation induced by nicotine and AngII in aged mice.

### 3.3. SP600125 Inhibits MMP-2 and MMP-9 Expression in Abdominal Aortic Lesions

**Figure 3:**
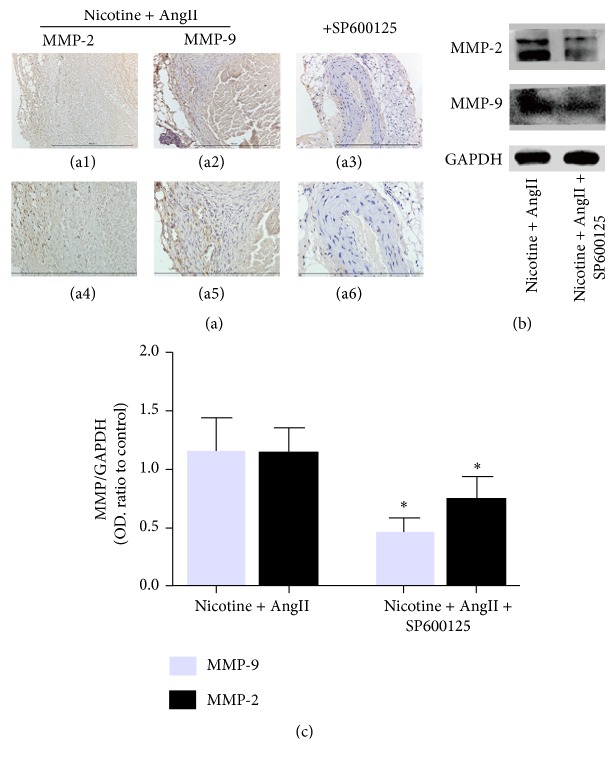
SP600125 suppresses the production of MMP-2 and MMP-9 in aortic tissue. (a) Representative images of immunohistochemical staining showing that MMP-2 and MMP-9 proteins are abundant in nicotine plus AngII-induced AAA tissues (a1, a2, a4, and a5), where they appear to be localized in the medial and outer smooth muscle cells. SP600125 downregulated the production of MMP-2 and MMP-9 in AAA lesions (a3 and a6). (b, c) Representative western blots for MMP-2 and MMP-9 revealing that SP600125 inhibits the expression of MMPs. The band optical density (OD) values (mean ± SD) of MMP-2 and MMP-9 were evaluated with ImageJ. GAPDH was used as an internal control and results are from independent triplicate experiments. *∗* refers to *P* < 0.05 versus coadministration.

To evaluate whether SP600125 inhibits the expression of MMP-2 and MMP-9 via the JNK pathway in abdominal aortic tissue, we applied immunohistochemical staining and western blotting to abdominal aortic tissue samples. Although MMP-2 and MMP-9 could be detected in both the nicotine plus AngII-treated and the SP600125-supplemented groups, the staining signal was significantly higher in tissue samples from mice treated with nicotine plus AngII, especially in the area around the site of rupture of the aortic media (Figure 3(a)). This finding indicated that the expression of MMP-2 and MMP-9 positively relates to the incidence of AAA and was inhibited by SP600125. Western blot analysis showed that, compared to the nicotine plus AngII group, the band intensities for MMP-2 and MMP-9 in the cotreatment plus SP600125 group were obviously lower (*P* < 0.05) (Figures 3(b) and 3(c)), corroborating the immunohistochemical staining results.

### 3.4. SP600125 Inhibits MCP-1 and RANTES Expression in Abdominal Aortic Lesions

**Figure 4:**
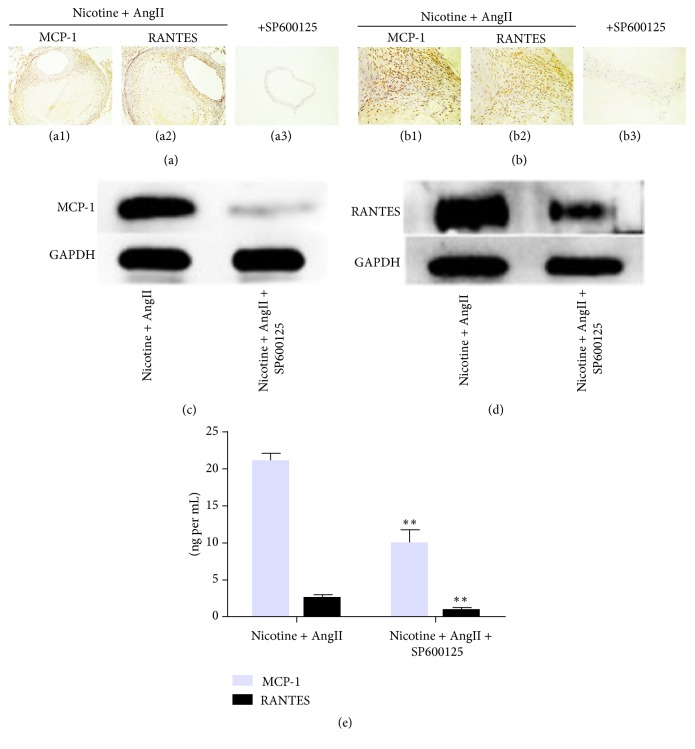
SP600125 suppresses the protein expression of chemokines in aortic tissue. (a) Representative transverse sections of mouse aortic tissue obtained after transient perfusion with nicotine plus AngII, with or without SP600125. MCP-1 and RANTES proteins were undetectable in normal aorta but were abundant in nicotine plus AngII-induced AAA tissues (a1 and a2), where they appear to be expressed in the medial and outer smooth muscle cells. SP600125 inhibited the protein expression of MCP-1 and RANTES in AAA lesions (a3). (c, d) Representative western blots for MCP-1 and RANTES. The band optical density (OD) values (mean ± SD) of MCP-1 and RANTES were evaluated using ImageJ. GAPDH was used as an internal control and results are from independent triplicate experiments. Chemokine expression in the serum as detected by ELISA (e). ^*∗∗*^ *P* < 0.01 versus coadministration.

Although chemokine expression is negligible in normal, unperfused aorta [15], MCP-1 and RANTES expression in abdominal aortic lesions increased remarkably in the group treated with nicotine plus AngII as compared with the saline-treated control group (Figures 4(a) and 4(b)). Both proteins appeared to be localized in the medial and outer smooth muscle cells. However, SP600125 supplementation suppressed the increase in protein expression of MCP-1 and RANTES induced by nicotine plus AngII as indicated by immunohistochemistry. These findings were supported by western blotting and ELISA results (Figures 4(c), 4(d), and 4(e)).

### 3.5. Nicotine Induces MCP-1 and RANTES Expression in MOVAS Cells in a Dose-Dependent Manner

**Figure 5:**
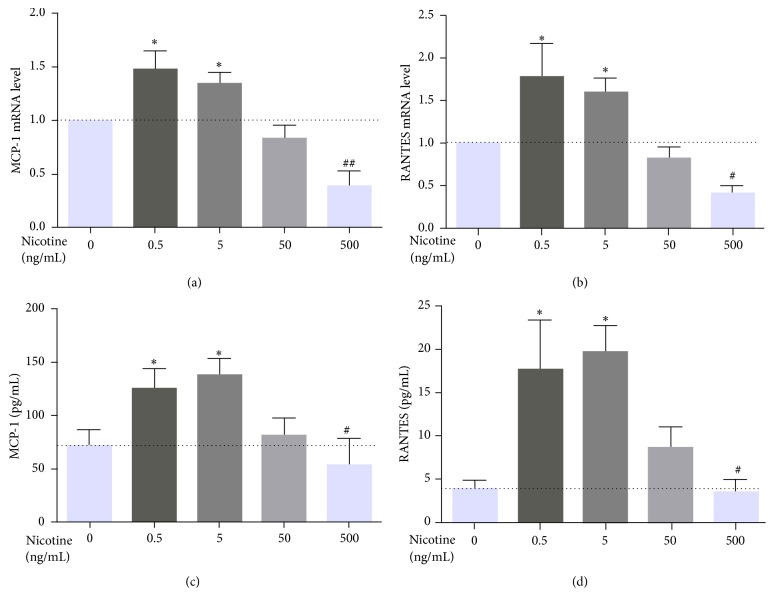
Expression of MCP-1 and RANTES under a concentration gradient of nicotine in MOVAS cells. Cellular mRNA expression levels of MCP-1 and RANTES (a, b) and the levels of secreted MCP-1 and RANTES in the supernatant (c, d) are shown. MCP-1 and RANTES expression as well as secretion was induced by nicotine in a dose-dependent fashion. The strongest expression was observed for 0.5 ng/mL and 5 ng/mL nicotine. Data are from independent triplicate experiments. ^*∗*^ *P* < 0.05 and ^*∗∗*^ *P* < 0.01 versus control; ^#^ *P* < 0.05 and ^##^ *P* < 0.01 versus the group treated with 5 ng/mL nicotine.

In our previous study, nicotine regulated the expression of MMP-2, MMP-9, and VCAM-1 in a concentration-dependent manner in MOVAS cells and RAW264.7 cells [16]. To assess whether nicotine stimulates vascular smooth muscle cells to express MCP-1 and RANTES in a dose-dependent fashion, MOVAS cells were exposed to nicotine at various concentrations (0, 0.5, 5, 50, and 500 ng/mL) for 3 h. Both qRT-PCR (Figures 5(a) and 5(b)) and ELISA (Figures 5(c) and 5(d)) showed that the strongest MCP-1 and RANTES expression was induced by 0.5 and 5 ng/mL nicotine, while expression was reduced following treatment with 50 ng/mL and a significant inhibitory effect was noted at 500 ng/mL.

### 3.6. SP600125 Suppresses Nicotine-Induced Upregulation of MCP-1 and RANTES in MOVAS Cells

**Figure 6:**
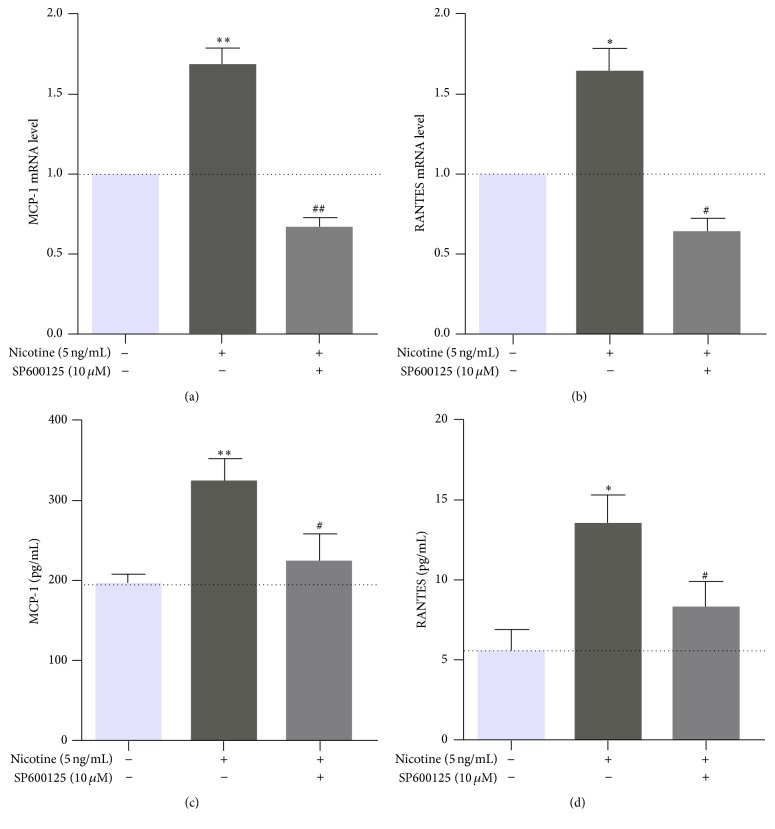
Influence of SP600125 on nicotine-induced expression of MCP-1 and RANTES in MOVAS cells. Cellular mRNA expression levels of MCP-1 and RANTES (a, b) and the levels of secreted MCP-1 and RANTES in the supernatant (c, d) are shown. Nicotine at 5 ng/mL could significantly upregulate the cellular mRNA expression as well as secretion of MCP-1 and RANTES, while SP600125 eliminated this effect. Data are from independent triplicate experiments. ^*∗*^ *P* < 0.05 and ^*∗∗*^ *P* < 0.01 versus control; ^#^ *P* < 0.05 and ^##^ *P* < 0.01 versus the group treated with 5 ng/mL nicotine.

To determine whether the upregulation of MCP-1 and RANTES induced by 0.5 ng/mL nicotine is regulated by JNK, MOVAS cells were pretreated with 10 *μ*M SP600125 for 30 min followed by exposure to 5 ng/mL nicotine for 3 h. RT-PCR showed that 10 *μ*M SP600125 suppressed the nicotine-induced upregulation of MCP-1 and RANTES (Figures 6(a) and 6(b)). Furthermore, ELISA showed that the nicotine-induced secretion of MCP-1 and RANTES was suppressed following treatment with 10 *μ*M SP600125 (Figures 6(c) and 6(d)). These results indicated that JNK is involved in the nicotine-induced expression and secretion of MCP-1 and RANTES in MOVAS cells.

### 3.7. SP600125 Attenuates Macrophage Migration by Inhibiting Vascular Smooth Muscle Cell Production of CC Chemokines

**Figure 7:**
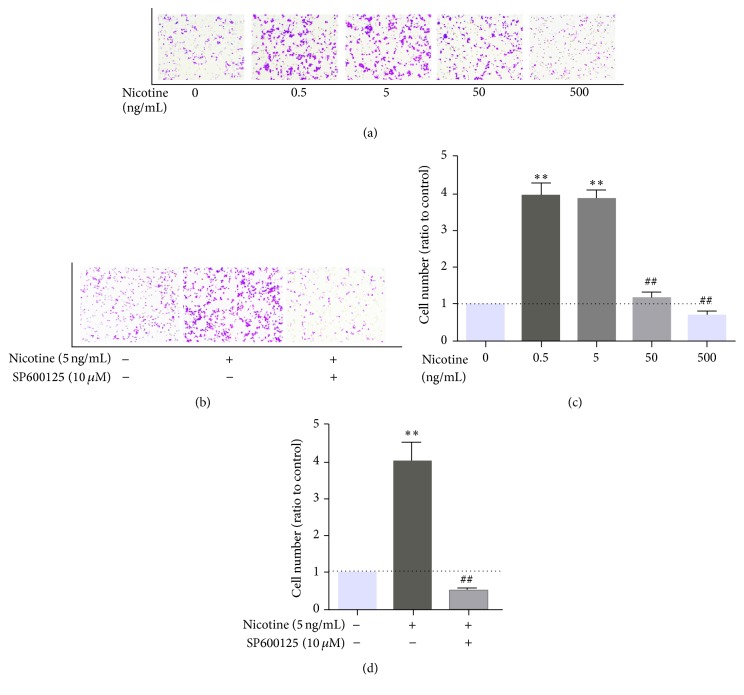
Transwell assay of macrophage migration. Representative pictures are shown. (a, b) Macrophages that had migrated to the lower chamber were counted after DAPI staining. (c, d) Bar graphs representing quantification of macrophage migration. Nicotine at 0.5 ng/mL or 5 ng/mL induced the strongest macrophage migration, while lower migration was observed following treatment at 50 ng/mL and a significant inhibitory effect was noted at a 500-ng/mL concentration. SP600125 inhibited the migration of macrophages. ^*∗*^ *P* < 0.05 and ^*∗∗*^ *P* < 0.01 versus control; ^#^ *P* < 0.05 and ^##^ *P* < 0.01 versus the group treated with 5 ng/mL nicotine.

Nicotine stimulated the expression of MCP-1 and RANTES in MOVAS cells, which is essential for macrophage migration. To investigate whether nicotine has an effect on macrophage migration by regulating the expression of MCP-1 and RANTES in MOVAS cells, a Transwell migration assay was conducted. MOVAS cells were exposed to different concentrations of nicotine (0, 0.5, 5, 50, and 500 ng/mL). Consistent with our previous findings, qRT-PCR and ELISA showed that the strongest MCP-1 and RANTES expression was induced by 0.5 and 5 ng/mL nicotine, with lower expression at 50 ng/mL and significant inhibition at 500 ng/mL (Figures 7(a) and 7(c)). A similar dose-dependent stimulatory effect of nicotine on macrophage migration was observed. However, pretreatment of the MOVAS cells with SP600125 suppressed the nicotine-stimulated macrophage migration (Figures 7(b) and 7(d)).

## 4. Discussion

Although the ability of nicotine to induce the development of AAA in ApoE^−/−^ mice model has been proved [4, 5] and AngII alone was able to induce the AAA formation in ApoE^−/−^ mice [17, 18], a more physiologically relevant experimental model without the interference of lipid metabolism is necessary and the underlying mechanisms remain to be studied. In this study, we chose to coadminister nicotine with a low dose of AngII by infusion in aged, wild-type C57BL/6J mice for 28 days. The results showed that nicotine or AngII alone did not induce AAA formation, while nicotine plus AngII did. Moreover, coadministration significantly enlarged the aortic diameter. Further, SP600125 supplementation significantly attenuated the AAA formation and aortic diameter dilation induced by nicotine plus AngII.

According to previous studies, either nicotine or AngII facilitates the formation and development of AAA, which seems to contradict our experimental results. Wang et al. demonstrated that 1 or 5 mg/kg/d of nicotine induced the development of aortic aneurysms in ApoE-deficient mice [19]; however, compared to ApoE-deficient mice, the incidence of AAA in C57BL/6J is much lower. Moreover, Zhang and Ramos found that 0.72 mg/kg/d of AngII induced AAA formation, but only in 2 out of 12 male C57BL/6J mice (6 to 8 months old) [6]; this difference may be explained by the smaller number of animals used, age distinction, or individual differences. Therefore, the different results may arise from the use of different animal models, the low dose used in this study, or individual differences.

The formation or rupture of AAA is a result of the concerted action of aortic wall inflammation and decreased medial smooth muscle cells [20]. MMPs, a family of zinc-dependent endopeptidases, are the most responsible for ECM degradation, especially in AAA [21]. The expression of MMP-2 and MMP-9 not only affects the prevalence of AAA but also correlates positively with the diameter of the AAA [22]. Additionally, MMPs contribute to the infiltration of macrophages [23]. As mentioned before, our previous study revealed that the inhibition of JNK could suppress the production of MMP-2 and MMP-9* in vitro* [16]; however, the relationship between JNK and MMPs in the nicotine-related aneurysm model remained unclear. The results of our present study indicated that JNK inhibition significantly decreased the expression of MMP-2 and MMP-9 as well as the incidence of AAA, implying that JNK may take part in AAA formation by regulating the MMP expression.

Chemokines are a superfamily of small secreted proteins (8–16 kDa), which are characterized by specific motifs in their N-terminal amino acid sequences [24]. MCP-1 and RANTES are two representative proteins of the CC chemokine subfamily, and both proteins exert a broad spectrum of effects in inflammatory responses [25]. A previous study in an elastase-induced AAA model demonstrated that the early events in aneurysm formation involve the accumulation of inflammatory cells in the adventitia via either the recruitment of circulating monocytes or the proliferation of resident macrophages [9]. In addition, monocytes from AAA patients show greater adhesion and transmigration [26]; however, the underlying mechanism is unclear. Our results show that SP600125 inhibits the production of MCP-1 and RANTES in aorta tissue of AAA model, indicating that the JNK pathway may contribute to nicotine plus AngII-induced AAA by inhibiting the expression of MCP-1 and RANTES.

From our* in vitro* experiment, we concluded that nicotine induced MCP-1 and RANTES expression in MOVAS cells in a dose-dependent manner, which was consistent with our previous finding of nicotine dose-dependently inducing MMP-2, MMP-9, and VCAM-1 expression [16]. Thus, nicotine might stimulate DNA synthesis in and proliferation of endothelial cells at low concentrations (10^−8^ M, 1.62 ng/mL) but inhibit this process at higher concentrations (10^−6 ^M, 162 ng/mL) [26]. However, the exact mechanism is still unclear.

Our mouse model recapitulates a number of important features of AAA in older smokers, such as the aged artery and more real concentration of nicotine, thus providing a useful experimental model to investigate the molecular and cellular events underlying aneurysm degeneration. However, our study had some limitations. While coadministration of nicotine and AngII contributed to aortic rupture and aneurysm in the aged mice, we cannot make a conclusive statement on whether these results involved an addictive effect or interactions between nicotine and AngII. In addition, it is not clear whether SP600125 attenuated the development of AAA by acting on each of nicotine and AngII or through a destructive effect on the interaction between both molecules. Further research is warranted.

Taken together, the data indicate that JNK is a proximal signaling molecule in the pathogenesis of nicotine plus AngII-induced AAA, a chronic inflammatory disease characterized by disruption of the ECM. Inhibition of JNK can suppress the formation of AAA by downregulating the expression of RANTES, MCP-1, MMP-2, and MMP-9. With further research, JNK-targeted therapy may provide nonsurgical therapeutic options for AAA, a disease that frequently results in fatal outcome.
